# The Future Perspectives of Older Adults as Reflected Through Digital Photocollage

**DOI:** 10.1093/geroni/igaf122.348

**Published:** 2025-12-31

**Authors:** Talia Elkarif, Shoshi Keisari, Hod Orkibi, Silvia Piol, Giada Mola, Frieder Lang, Ines Testoni

**Affiliations:** University of Haifa, Haifa, HaZafon, Israel; University of Haifa, Haifa, HaZafon, Israel; University of Haifa, Haifa, HaZafon, Israel; University of Haifa, Haifa, HaZafon, Israel; Università degli Studi di Padova DPSS Department, Padova, Veneto, Italy; Friedrich-Alexander-Universität Erlangen-Nürnberg, Nuremberg, Bayern, Germany; Università degli Studi di Padova FISPPA Department, Section of Applied Psychology, Padova, Veneto, Italy

## Abstract

Future time perspective refers to our subjective perceptions of the future. These perspectives change across the lifespan, shaping our goals, and accordingly, our actions. Exploring future perspectives can support self-development while aging but may also evoke perspectives that are difficult to discuss. While the arts can provide a safe and creative environment for older adults to explore future time perspectives, studies in this field are scarce. The current qualitative study explored the future time perspectives of older adults as expressed through digital photocollage. This study was part of a larger project aimed at developing an online arts-based intervention for community-dwelling older adults. Twenty-four Italian and Israeli older adults aged 78–92 participated in a brief therapeutic online intervention integrating Dignity Therapy and digital photocollage. Visual and verbal data were analyzed using polytextual thematic analysis. Four themes emerged: three reflecting participants’ attitudes toward the future and one reflecting the evolution of future time perspectives during the creative process. The findings indicate that despite the short-term nature of the creative online intervention, and the complex topic at hand, participants expressed multiple future perspectives. The study also highlights the potential of digital and artistic methods to facilitate both the expression and the expansion of older adults’ future perspectives. These findings contribute to the growing literature on creative arts therapies in later life, demonstrating the potential of digital arts-based approaches in fostering meaningful engagement with future-oriented narratives among older adults.

